# Predicting the Potential Distribution of *Plantago asiatica* L. in Jiangxi Province Under Climate Change Scenarios Using the MaxEnt Model

**DOI:** 10.3390/plants15172732

**Published:** 2026-09-07

**Authors:** Junxia Yan, Jiangkun Zheng, Jianfeng Zhang

**Affiliations:** 1 Department of Geography, Handan University, Handan 056005, China; yjx_junxia@126.com (J.Y.);; 2 Forest Ecology and Conservation in the Upper Reaches of the Yangtze River Key Laboratory of Sichuan Province, College of Forestry, Sichuan Agricultural University, Chengdu 611130, China

**Keywords:** MaxEnt model, climate change, potential suitable habitat, centroid migration, Jiangxi Province

## Abstract

*Plantago asiatica* L. is an important medicinal and edible plant in China, whose wild resources have declined sharply due to overharvesting. Accurately assessing its potential suitable habitats and identifying the dominant environmental factors are of great scientific significance for germplasm conservation. In this study, we focused on Jiangxi Province and, based on 79 field occurrence records and 29 environmental variables, employed a MaxEnt model optimized using the *kuenm* package (version 1.1.9) (AUC = 0.806) to simulate the potential distribution of this species under current and three future Shared Socioeconomic Pathway (SSP) scenarios (SSP126, SSP370, and SSP585). The results indicated that static non-climatic factors at the regional scale—specifically, soil clay content (contribution rate: 56.7%)—were the dominant force determining the distribution of *Plantago asiatica*, with an influence far exceeding that of climatic and topographic factors. Under all future SSP scenarios, its distribution range exhibited notable spatiotemporal conservatism, and the centroid showed no long-distance migration. This study provides a scientific reference for optimizing the conservation framework of *Plantago asiatica* germplasm resources, identifying priority cultivation areas, and formulating regional adaptive management strategies.

## 1. Introduction

Against the backdrop of severe challenges to global biodiversity conservation and sustainable resource utilization, precisely assessing the responses of plant species with significant ecological and economic value to climate change has become a central issue in conservation biology and ecology [1,2]. Jiangxi Province, serving as a major ecological barrier in southern China, exhibits a spatial pattern of ecological environment quality characterized by “superior in mountainous and hilly areas, and moderate in plains” [3]. Medicinal plants, as critical strategic resources for safeguarding human health, are facing dual threats from global warming and habitat fragmentation [4]. *Plantago asiatica* L., a perennial herbaceous species in the genus *Plantago* (Plantaginaceae), is a traditional bulk Chinese medicinal material. Its whole plant possesses diuretic, heat-clearing, and detoxifying properties, serving both medicinal and edible purposes, with substantial market demand [5,6]. However, long-term indiscriminate over-harvesting has led to a sharp decline in wild *Plantago asiatica* resources and severely impeded natural population regeneration [7]. Therefore, accurately revealing the potential suitable habitat distribution and spatiotemporal dynamics of *Plantago asiatica* under ongoing climate change is a critical scientific question that urgently needs to be addressed for developing strategies for resource conservation, artificial domestication and cultivation, and sustainable utilization.

Species distribution suitability assessment delineates spatial patterns of suitability by analyzing the congruence between species requirements and environmental conditions [8]. Traditional evaluation methods (e.g., analytic hierarchy process, fuzzy mathematics, weighted scoring [9,10,11]) suffer from limitations including insufficient objectivity in data processing and poor flexibility in variable selection [12]. The integration of GIS technology has enhanced quantitative analytical capabilities [13], yet this remains inadequate for handling complex environmental relationships and dynamic predictions. Species distribution models (SDMs) integrate species occurrence data with environmental factors to simulate niche requirements and predict potential habitats [14]. Common SDM algorithms include Maximum Entropy (MaxEnt), Generalized Additive Models (GAMs), Generalized Linear Models (GLMs), and Random Forest Models (RFMs) [14,15]. Among these, MaxEnt has been widely applied due to its high predictive accuracy, operational simplicity, ability to handle small sample sizes, and reliance solely on occurrence data [16,17,18]. MaxEnt has expanded from predicting wildlife habitats [19,20] to forecasting potential cultivation areas for crops [21,22].

When modeling species’ responses to future climate change scenarios, the reliability of model results critically depends on the accurate quantification of multi-dimensional environmental variables and the appropriate optimization of model parameters. Although climatic factors—particularly precipitation in the driest quarter, temperature seasonality, and extreme low temperatures—are the dominant drivers of species distributions at broad scales [23,24], non-climatic factors such as soil physicochemical properties and topographic features also play non-negligible roles in characterizing suitable habitats at regional scales [25]. Consequently, relying solely on climatic factors may introduce prediction biases, which are especially pronounced at regional scales: as the study area narrows to regions with relatively homogeneous climatic conditions, the constraining effects of non-climatic factors on species distributions may surpass those of climatic factors.

Jiangxi Province, as one of the important distribution areas and traditional production regions for *Plantago asiatica* in China, is facing dual pressures from habitat fragmentation and urbanization. The spatial pattern of its suitable habitats remains unclear, and the dynamic responses to future climate change have yet to be elucidated. In terms of distribution prediction, Liao et al. [26] revealed that precipitation and human activities are the dominant drivers shaping the distribution of the genus Plantago at the national scale. However, whether these findings are applicable to Jiangxi Province—a subtropical humid region—remains uncertain. Therefore, conducting a prediction of the potential suitable habitat of *Plantago asiatica* in Jiangxi Province can not only provide a scientific basis for the conservation of germplasm resources and the strategic layout of artificial cultivation bases, but also serve as a test case to identify the dominant limiting factors of potential suitable habitat at the regional scale.

In light of this, the present study focuses on Jiangxi Province, integrating climatic, soil, and topographic environmental predictors based on precisely georeferenced occurrence records of *Plantago asiatica*, and employs the *kuenm* package to optimize key parameters of the MaxEnt model to achieve more robust predictions [27]. This study aims to address the following core objectives: (1) to identify the dominant environmental factors constraining the geographical distribution of *Plantago asiatica* at the regional scale and determine their optimal suitability thresholds; (2) to delineate the spatial pattern of potential suitable habitats under current climatic conditions; (3) to predict and compare the range shifts and centroid migration trajectories of suitable habitats under different future climate scenarios (SSP126, SSP370, and SSP585). The overarching goal is to provide scientific evidence for the conservation of *Plantago asiatica* germplasm resources and the planning of artificial cultivation in Jiangxi Province.

## 2. Results

### 2.1. Selection of Dominant Environmental Factors and Optimal Range Analysis

#### 2.1.1. Preliminary Screening of Key Factors Using Pearson’s Correlation

To avoid distortion in weight allocation and overfitting in the MaxEnt model caused by multicollinearity, Pearson’s correlation coefficients were applied to screen the initial environmental factors. Based on the percent contributions calculated from a preliminary MaxEnt model, variables with low contribution rates among highly correlated pairs (|r| ≥ 0.8) were excluded [28]. Based on the Pearson correlation analysis, 11 environmental factors were retained as preliminary key variables from the initial 29: bio1 (annual mean temperature), bio3 (isothermality), bio8 (mean temperature of the wettest quarter), bio14 (precipitation of the driest month), bio15 (precipitation seasonality), bio17 (precipitation of the driest quarter), bio18 (precipitation of the warmest quarter), bio19 (precipitation of the coldest quarter), alt (altitude), aspect, and clay (clay content).

#### 2.1.2. Identification of Key Environmental Factors Using the Jackknife Test

**Figure 1:**
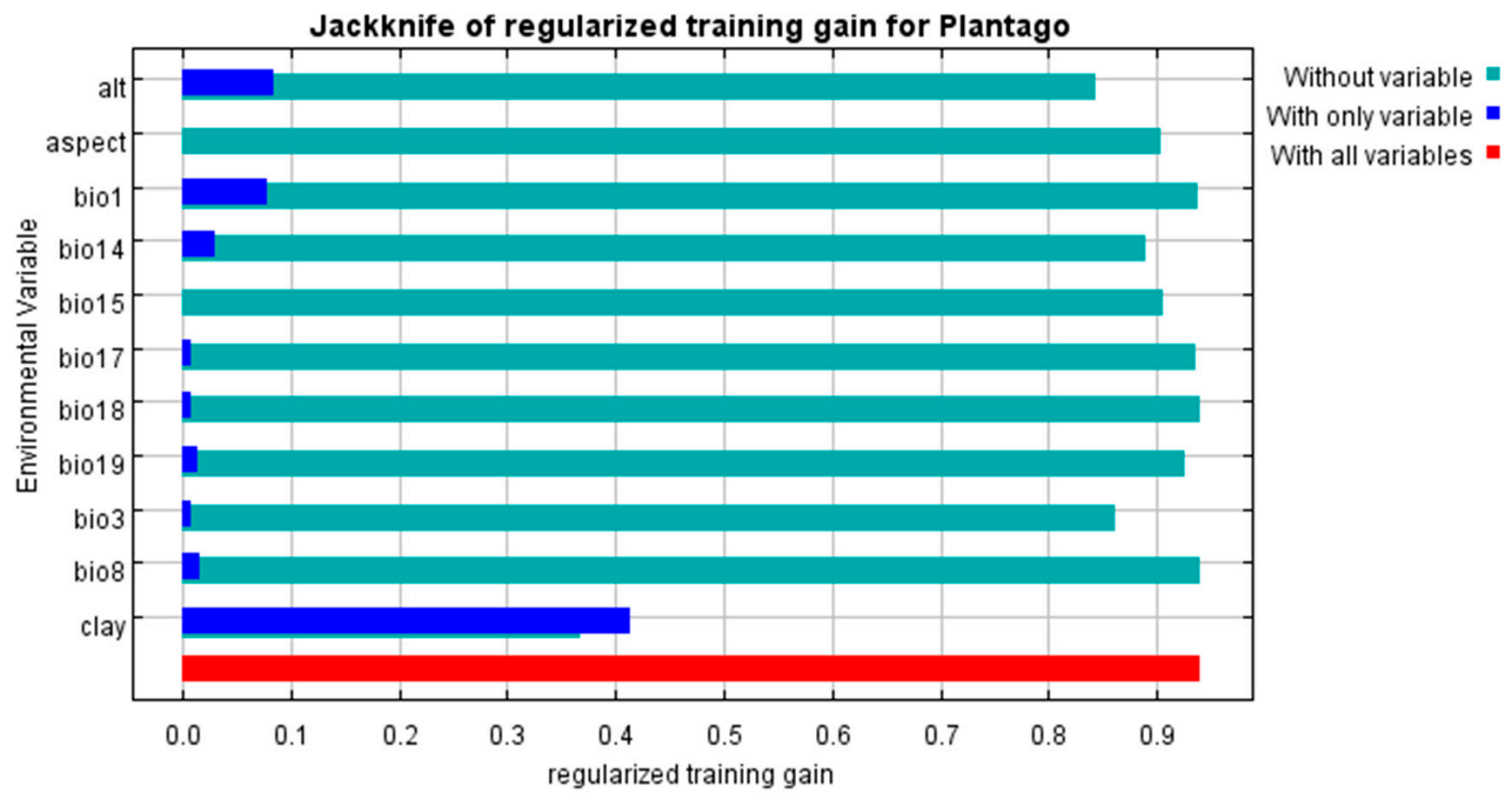
Regularized training gain of each environmental variable for the distribution of *Plantago asiatica* (jackknife test).

The jackknife test and variable importance evaluation identified the key environmental factors closely associated with the distribution of *Plantago asiatica* (Figure 1). The analysis revealed that clay content (clay) exhibited the most significant contribution, with both contribution rate (56.7%) and permutation importance (26.3%) ranking among the highest, and its independent predictive capability substantially exceeded that of other factors (Table 1). The secondary set of environmental variables comprised hygrothermal and topographic factors, including isothermality (bio3, 12.4%), altitude (alt, 6.5%), precipitation of the coldest quarter (bio19, 6%), and precipitation seasonality (bio15, 6%). Although the contribution rates of bio19 and bio15 were similar, their permutation importance differed: bio15 exhibited a higher permutation importance (6.4%) than bio19 (2.1%), indicating that precipitation seasonality possessed greater irreplaceability in model prediction. Despite the relatively low contribution rate of altitude (alt), its permutation importance was remarkably high (23.4%), rendering it highly irreplaceable in model prediction. The remaining variables (aspect, bio8, etc.) exhibited relatively low contribution rates and permutation importance, contributing only weak auxiliary effects. Consequently, clay, bio3, alt, bio19, and bio15 were identified as the key environmental factors constraining the distribution of *Plantago asiatica*.

#### 2.1.3. Optimal Range Analysis of Key Environmental Factors

**Figure 2:**
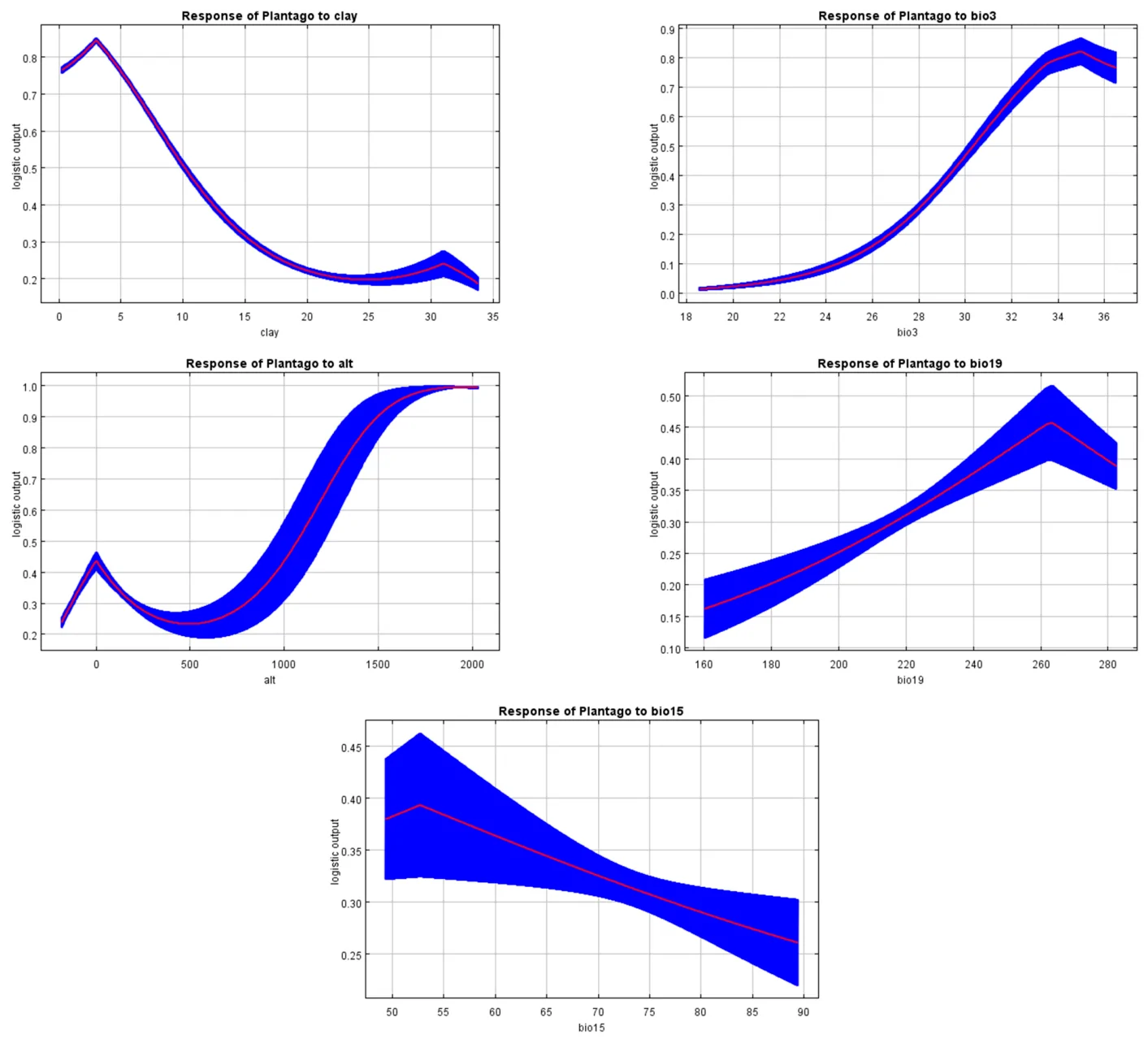
Response curves of key environmental variables.

The environmental response curves reflect the variation in the occurrence probability of *Plantago asiatica* along environmental gradients. Based on the marginal response curves generated by MaxEnt (Figure 2), the optimal ranges for each key environmental factor were determined [29,30,31,32]: for clay content, the occurrence probability generally showed a decreasing trend with increasing clay content, reaching a peak at extremely low levels, indicating a preference for loose, low-clay soils. For isothermality (bio3), the occurrence probability showed a continuously increasing trend, with the optimal range approximately between 31.2% and 36.7%. For altitude (alt), the occurrence probability exhibited a “V”-shaped response pattern, initially decreasing and then increasing with elevation [33]. Precipitation of the coldest quarter (bio19) showed a unimodal response, while precipitation seasonality (bio15) exhibited a decreasing trend in occurrence probability as the index increased. Collectively, the contribution of clay (56.7%) occupied an absolutely dominant position, rendering it the most critical limiting factor for regional distribution; bio3, alt, bio19, and bio15 jointly defined the suitable interval of this species under the synergistic effects of topography and hydrothermal conditions, reflecting the dual ecological adaptation strategy of *Plantago asiatica* to soil texture and climatic seasonality.

### 2.2. Model Performance Evaluation

**Figure 3:**
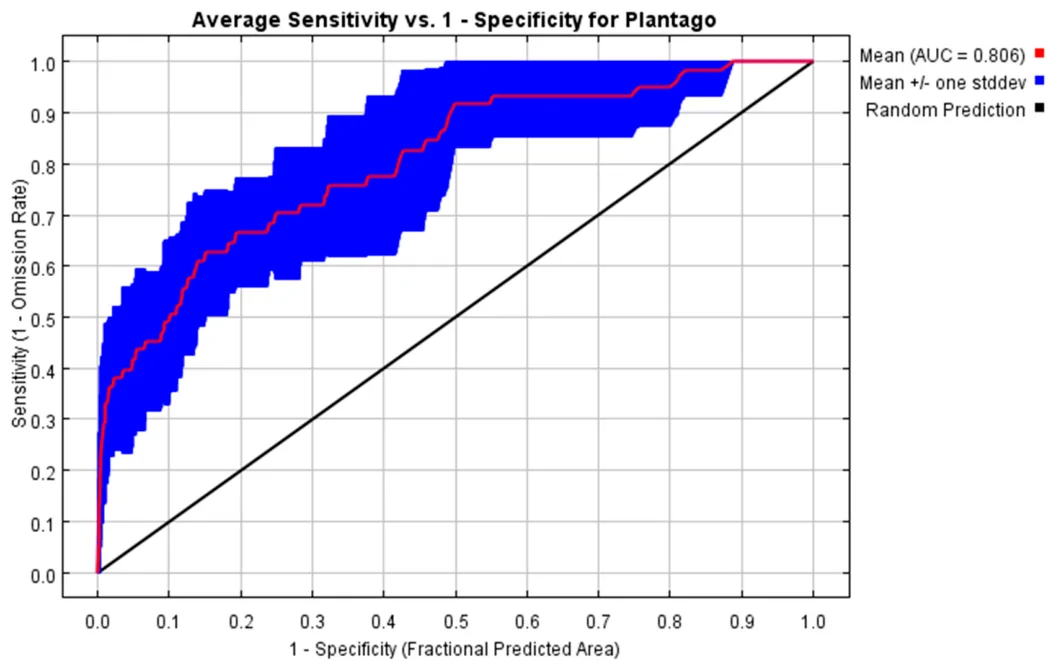
ROC curves and mean AUC for the *Plantago asiatica* distribution model.

The predictive accuracy of the MaxEnt model was evaluated using the area under the receiver operating characteristic curve (AUC) [34,35]. AUC values range from 0 to 1, with values closer to 1 indicating higher predictive accuracy. After running the MaxEnt model, the mean AUC value for the training dataset was 0.806 (with a standard deviation of 0.089) (Figure 3), indicating good predictive performance and high accuracy for modeling the distribution of *Plantago asiatica* [36,37,38].

### 2.3. Current Distribution of Ecological Suitable Habitats Under Contemporary Climate

The suitability predictions generated by the MaxEnt model inherently exhibit distributional patterns and natural variation. Different regions, owing to the combined effects of climatic, soil, and topographic factors, show varying degrees of suitability for *Plantago asiatica*, and these differences manifest as distribution patterns in the model outputs. In this study, the 10th percentile training presence logistic threshold output by MaxEnt was first used to distinguish suitable from unsuitable habitats, with areas below this threshold classified as unsuitable. On this basis, to reflect the gradient differences within suitable areas, the suitable areas were further divided into low, moderate, and high suitability categories, following the classification standards established in previous studies [39].

**Figure 4:**
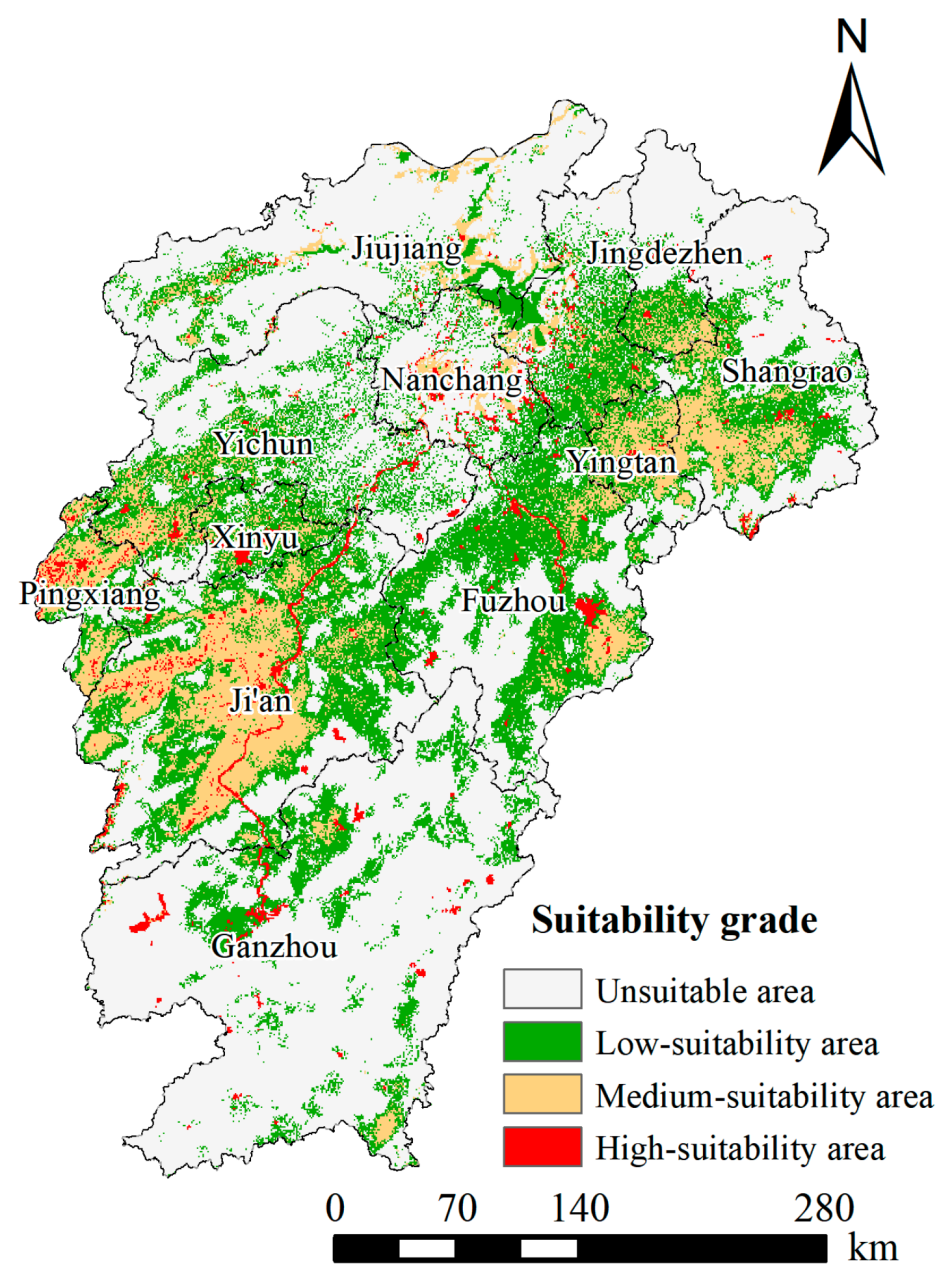
Current distribution map of suitable areas for *Plantago asiatica*.

The MaxEnt model results (Figure 4) revealed a significant spatial heterogeneity pattern of suitable habitats for *Plantago asiatica*. Highly suitable habitats occupied a relatively small area (approximately 0.39 × 10^4^ km^2^, accounting for 1.40%) and were mainly distributed as banded patches in the central-northern region of Jiangxi. Moderately suitable habitats (2.16 × 10^4^ km^2^, accounting for 14.77%) and low-suitability habitats (4.43 × 10^4^ km^2^, accounting for 34.51%) covered relatively large areas, primarily distributed as large contiguous patches across central and south-central Jiangxi, encompassing most of Ji’an City, Fuzhou City, Xinyu City, and Yingtan City. Unsuitable habitats accounted for the largest proportion (approximately 9.71 × 10^4^ km^2^, accounting for 49.32%), extensively covering the vast majority of Ganzhou City and most of the northern region of Jiujiang City. Additionally, unsuitable patches were sporadically distributed in western Yichun, Pingxiang City, the southern edge of Ji’an, and southwestern Fuzhou, as well as the northern edges of Nanchang, Shangrao, and Jingdezhen.

### 2.4. Distribution of Ecological Suitable Habitats Under Future Climate Change Scenarios

**Figure 5:**
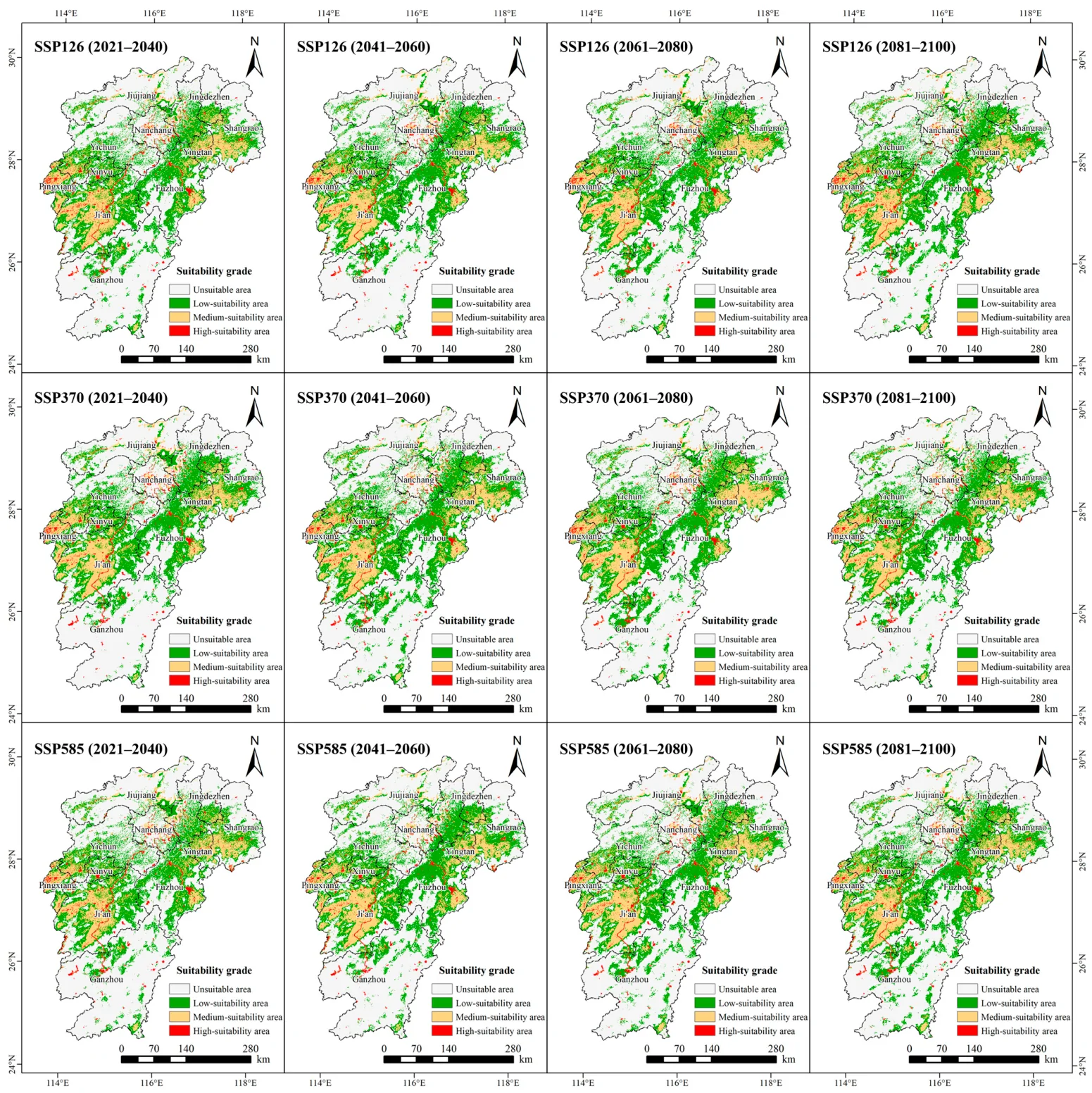
Spatial distribution patterns of potential suitable habitats for *Plantago asiatica* under future climate scenarios.

**Figure 6:**
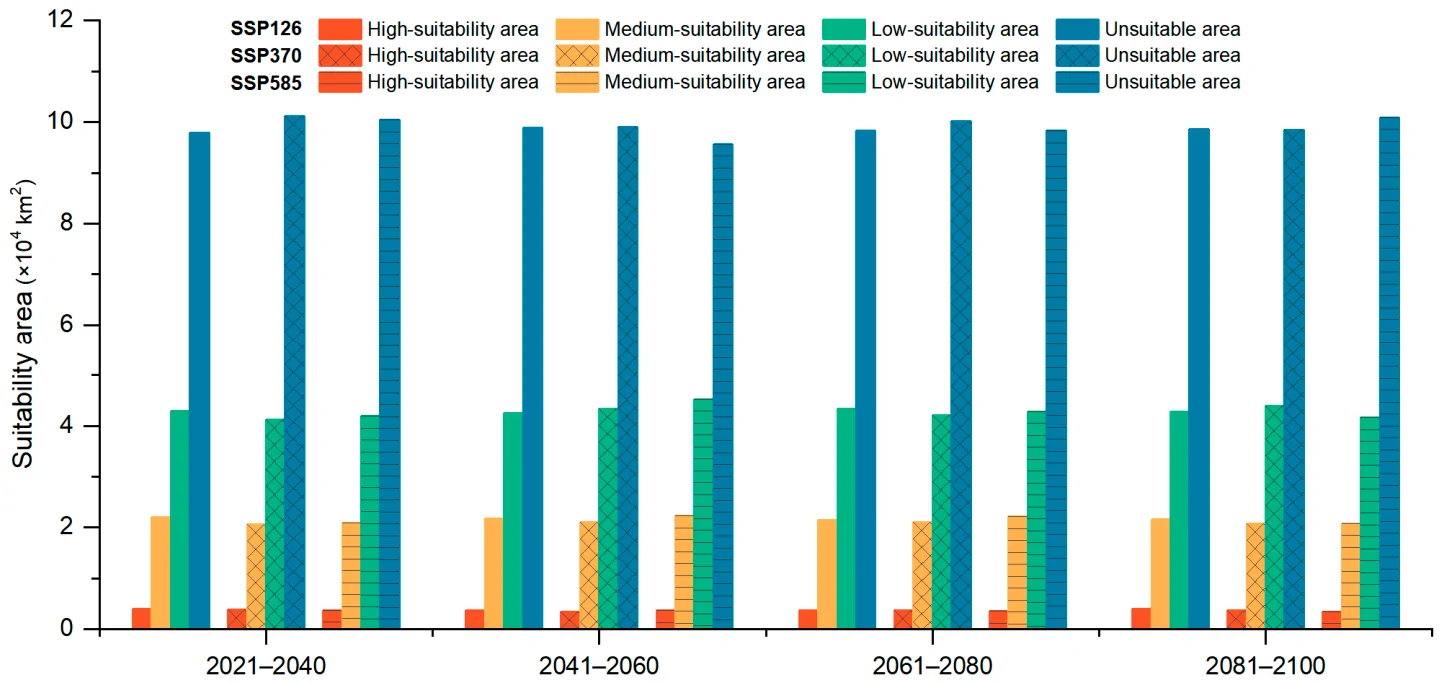
Areas of each suitability class for *Plantago asiatica* under the three different climate scenarios.

Based on the optimized MaxEnt model, this study simulated the dynamics of suitable habitats for *Plantago asiatica* under three SSP scenarios across four future time periods (Table 2, Figure 5). Overall, under the SSP126 scenario, the area of highly suitable habitats initially decreased and then stabilized, returning to the baseline level by 2081–2100. Under the SSP370 scenario, the area of highly suitable habitats decreased progressively over time, reaching its lowest value across all periods in 2081–2100. Under the SSP585 scenario, the area of highly suitable habitats exhibited a fluctuating pattern of initial decline followed by recovery, with a greater amplitude of fluctuation than that under SSP126. Unsuitable habitats maintained a relatively large proportion under all three scenarios, with an increase in area during the later period of the SSP370 scenario. Relative to the historical baseline period, no significant net expansion of highly suitable habitats was observed under any scenario, a result that markedly contrasts with the widely recognized trend of significant expansion of medicinal plant suitable habitats driven by climate warming at the national scale (Figure 6).

Under the SSP126 scenario, the area of highly suitable habitats remained at 0.40 × 10^4^ km^2^ (2.38%) during 2021–2040, slightly decreased to 0.38 × 10^4^ km^2^ (2.26%) during 2041–2060, maintained this level during 2061–2080, and then recovered to 0.40 × 10^4^ km^2^ (2.38%) during 2081–2100. Moderately suitable habitats remained relatively stable, ranging between 2.15 × 10^4^ km^2^ and 2.21 × 10^4^ km^2^. The area of low-suitability habitats was generally maintained between 4.25 × 10^4^ km^2^ and 4.33 × 10^4^ km^2^, while unsuitable habitats also remained within a stable range of 9.79 × 10^4^ km^2^ to 9.89 × 10^4^ km^2^. Spatially, highly suitable habitats remained persistently concentrated in the central-northern region of Jiangxi (the transitional zone of Nanchang, Yichun, Xinyu, and Fuzhou), with no significant displacement of their patches across the four time periods.

Under the SSP370 scenario, the area of each suitability class exhibited significant spatial redistribution over time. The area of highly suitable habitats progressively decreased from 0.38 × 10^4^ km^2^ during 2021–2040, reaching its lowest value of 0.35 × 10^4^ km^2^ across all periods in 2081–2100; spatially, the edges of the core patches of highly suitable habitats continuously contracted, but the main body did not shift. The area of moderately suitable habitats increased to 2.23 × 10^4^ km^2^ during 2041–2060 and then decreased to 2.08 × 10^4^ km^2^ by the end of the period. The area of unsuitable habitats significantly increased in the later period, rising from 9.83 × 10^4^ km^2^ during 2061–2080 to 10.09 × 10^4^ km^2^ during 2081–2100. Spatially, unsuitable habitats were primarily distributed in southern, northern, and western Jiangxi.

Under the SSP585 scenario, the area of highly suitable habitats decreased from 0.39 × 10^4^ km^2^ (2.31%) during the initial period (2021–2040) to the lowest value of 0.35 × 10^4^ km^2^ (2.07%) across all periods during 2041–2060 and then recovered to 0.38 × 10^4^ km^2^ (2.28%) during 2081–2100. Spatially, highly suitable habitats exhibited local contraction in the central-northern core region of Jiangxi during the initial period, but re-expanded after 2061–2080, with their extent not fully recovering to the initial level. The area of moderately suitable habitats increased from 2.07 × 10^4^ km^2^ during 2021–2040 to a peak of 2.11 × 10^4^ km^2^ during 2041–2060 and then decreased to 2.08 × 10^4^ km^2^ by the end of the period. Spatially, moderately suitable habitats formed contiguous bands stably extending from north-central Ji’an to most of Fuzhou, without positional shifts or fragmentation.

### 2.5. Centroid Migration Analysis of Highly Suitable Habitats for Plantago asiatica

**Figure 7:**
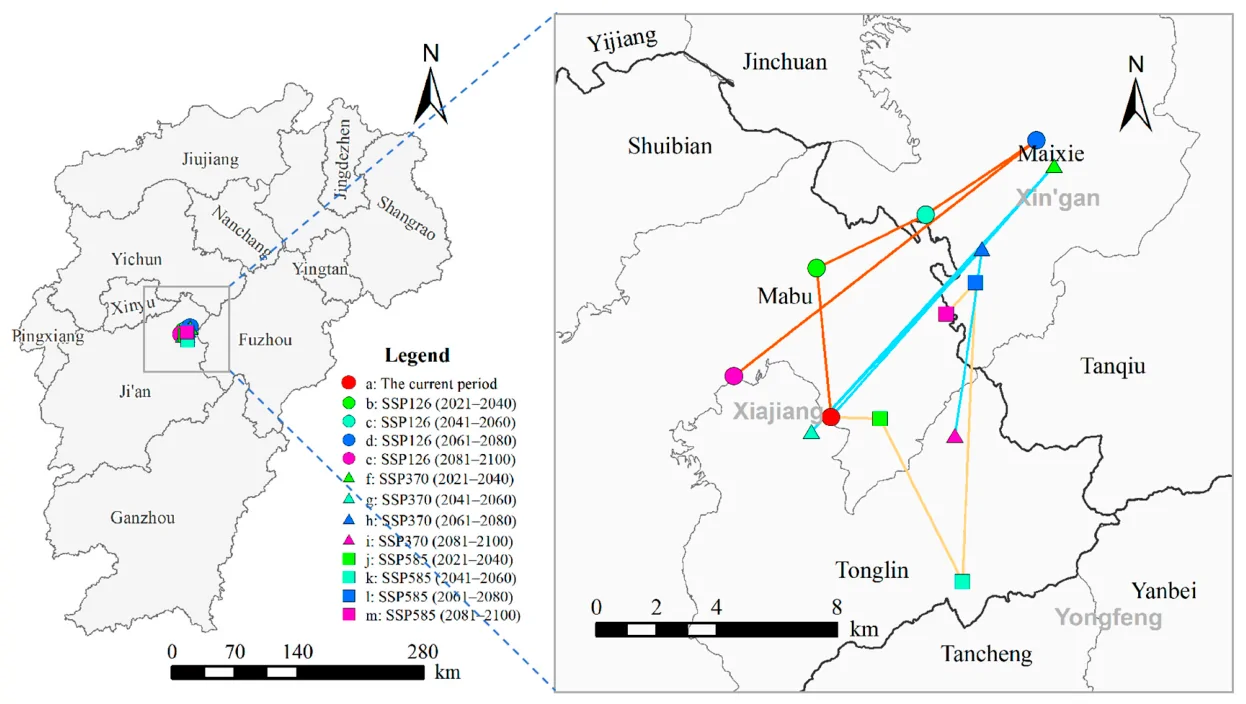
Centroid migration trajectories of highly suitable habitats for *Plantago asiatica* under different climate scenarios.

Under different carbon emission scenarios, the distribution centroid of *Plantago asiatica* in Jiangxi Province exhibited a consistent pattern of local oscillation and back-and-forth movement (Figure 7, Table 3). The current potential suitable habitat is located within Mabu Town, Xiajiang County, Ji’an City. Under the SSP126 scenario, the centroid exhibited a northward-then-southward migration pattern: from 2021–2040, it moved 4.98 km north from Mabu Town; from 2041–2060, it moved 3.97 km northeast to Maixie Town; from 2061–2080, it continued to move 4.37 km northeast; and by 2081–2100, it moved 12.74 km back southwest from Maixie Town to Mabu Town. Under the SSP370 scenario, the centroid exhibited a back-and-forth oscillation pattern: from 2021–2040, it moved 10.67 km northeast to Maixie Town; from 2041–2060, it moved 11.03 km southwest to Tonglin Township; from 2061–2080, it moved 7.49 km northeast again to Maixie Town; and from 2081–2100, it moved 6.25 km south to the northeastern part of Tonglin Township. Under the SSP585 scenario, the centroid exhibited a circular detour pattern: from 2021–2040, it moved 1.63 km east; from 2041–2060, it moved 5.89 km southeast to Tonglin Township; from 2061–2080, it moved sharply 9.99 km north to Maixie Town; and by 2081–2100, it moved 1.40 km back southwest. In summary, under continued climate change, the potential highly suitable habitats of *Plantago asiatica* did not exhibit significant expansion or directional migration, and the centroid remained within the vicinity of Mabu Town, Maixie Town, and Tonglin Township. This highly stable centroid dynamic provides a spatial reference for the long-term and stable site selection of artificial cultivation bases in the central-northern core region of Jiangxi.

## 3. Discussion

### 3.1. Model Performance Evaluation and Dominant Environmental Factor Analysis

In this study, following parameter optimization using the *kuenm* package (FC = LQP, RM = 0.7), the MaxEnt model achieved an AUC value of 0.806, indicating good predictive accuracy. This value is slightly lower than the AUC (0.90) reported in the national-scale study by Liao et al. [26], reflecting the increased difficulty of model fitting due to compressed environmental gradients at regional scales. The intricate topography of Jiangxi Province, characterized by interlocking mountains, hills, and plains, combined with the significant microclimatic regulation exerted by Poyang Lake, increases the challenge of distinguishing suitable from unsuitable habitats. Nevertheless, the optimized MaxEnt model effectively extracted the dominant signals governing *Plantago asiatica* distribution. Through integrated Pearson’s correlation analysis, contribution rate ranking, and the jackknife test, five key factors were identified—clay content (clay), altitude (alt), isothermality (bio3), precipitation of the coldest quarter (bio19), and precipitation seasonality (bio15). Among these, clay content exhibited the highest contribution rate (56.7%), exceeding the combined contribution of climatic and topographic factors, underscoring the central constraining role of soil texture.

These findings contrast with the national-scale study by Liao et al. [26], which identified precipitation and human activities as dominant drivers, whereas the core constraining factor in the present study is soil texture, with soil factors exerting constraints that surpass climatic influences. This discrepancy reflects a scale-dependent effect [40]: at the national scale, hydrothermic gradients represent the primary limitation, whereas within the climatically relatively homogeneous subtropical humid region of Jiangxi Province, the bottleneck has shifted from “precipitation availability” to the micro-scale issue of “whether soil texture is suitable for root penetration”. This finding precisely underscores the necessity of conducting regional-scale potential suitable habitat studies for *Plantago asiatica*.

The predominance of clay content can be mechanistically explained by the matching relationship between soil characteristics and root architecture. The Quaternary red clay-derived soils widely distributed in north-central Jiangxi are prone to compaction and exhibit low porosity, while *Plantago asiatica*, as a fibrous-rooted shallow-rooted plant, is highly sensitive to the permeability of the topsoil layer, resulting in a monotonic decline in occurrence probability with increasing clay content [41]. To verify the species-specificity of soil texture constraints, we compared another authentic medicinal material from Jiangxi, Ligusticum sinense (Chuanxiong), which possesses deeper root systems and exhibits an insensitive response to surface soil texture, with soil factors contributing only approximately 13.5% [42], forming a stark contrast with *Plantago asiatica*.

### 3.2. Multi-Dimensional Drivers of the Current Distribution Pattern

Under current climatic conditions, the suitable habitats of *Plantago asiatica* exhibit a pattern characterized by high suitability in the central region and unsuitability in the northern and southern areas. Highly suitable habitats occupy only 0.39 × 10^4^ km^2^, distributed as banded patches in the central-northern region of Jiangxi. Moderately suitable habitats (2.16 × 10^4^ km^2^) are extensively distributed as contiguous patches in central and northeastern Jiangxi. Low-suitability habitats (4.43 × 10^4^ km^2^) surround the aforementioned areas, extensively covering eastern, northeastern, and the marginal areas of south-central Jiangxi. Unsuitable habitats cover an area of 9.71 × 10^4^ km^2^, spatially predominantly occupying the northern and southern regions of Jiangxi.

This pattern is jointly governed by three factors: macro-geomorphology, local topography and hydrology, and soil physicochemical properties. Southern Jiangxi lies on the remnants of the Nanling Mountains, where higher elevations and insufficient thermal resources prevail. The optimum growth temperature for *Plantago asiatica* is 20–24 °C, and growth is inhibited when temperatures exceed 32 °C. Although northern Jiujiang has suitable thermal conditions, the lakeside plain soils of Poyang Lake are excessively sandy, with weak water and nutrient retention capacity. The Ji’an and Fuzhou areas of central Jiangxi have moderate elevations and favorable hydrothermic conditions, which precisely fall within the suitable range for *Plantago asiatica*. As a major dao-di production area for Plantaginis Semen in China, Jiangxi accounts for over 70% of national production, with the Sanhu Town of Xingan County—a core production area—maintaining a stable cultivation area of 667–800 ha. This actual production layout is highly consistent with the spatial distribution of highly suitable habitats predicted by the model, exhibiting strong spatial consistency and providing important field-based corroboration for the model predictions. At a finer scale, the regional distribution of clay content (contribution rate: 56.7%) further explains the spatial localization of highly suitable habitats—soils with low clay content and moderate texture are the core limiting factors for the formation of highly suitable habitats.

The national-scale study by Liao et al. [26] demonstrated that human activities can explain over 50% of the distribution of the genus *Plantago*. In the present study, high-suitability areas were mainly concentrated in the urban fringes and peri-urban agricultural zones of the central-northern region of Jiangxi. This discrepancy arises because at the national scale, human activities generally encroach upon habitats, whereas abandoned farmlands, orchards, and roadside moist zones in these urban fringes coincidentally simulate the moist, low-competition environments preferred by *Plantago asiatica*, effectively functioning as “alternative habitats”. Peri-urban transition zones should not be overlooked simply because they are adjacent to cities; rather, they should be incorporated into priority conservation areas [43].

### 3.3. Suitable Habitat Evolution Under Future Climate Change Scenarios

Under all three future SSP scenarios, the area of highly suitable habitats for *Plantago asiatica* did not exhibit a large-scale expansion trend but instead showed a high degree of spatiotemporal conservatism. Under the SSP370 and SSP585 scenarios, the area of highly suitable habitats reached its lowest value (0.35 × 10^4^ km^2^) during 2081–2100 and 2041–2060, respectively; under the SSP126 scenario, it exhibited slight fluctuations and returned to the baseline level. This non-monotonic variation reflects the complex interactions between warming and seasonal precipitation changes, with static soil physicochemical properties (clay content) at the regional scale largely buffering the driving effects of climate change. This trend markedly contrasts with the widely recognized notion at the national scale that “climate change is significantly altering species distribution ranges” [44,45,46]. Not all plant taxa exhibit expanding trends [23]; different plant groups show fundamentally divergent responses to climate change, influenced by both ecological amplitude and dispersal capacity [45].

The stability of highly suitable habitat area does not equate to synchronous growth in actual distribution. Centroid migration analysis revealed that under all three scenarios, the centroid of highly suitable habitats did not undergo long-distance directional migration and remained within the vicinity of Mabu Town, Maixie Town, and Tonglin Township. This cross-scenario consistency provides clear guidance for long-term site selection of cultivation bases. The limited fluctuations in centroid trajectories are associated with changes in precipitation patterns; when precipitation in the driest month decreases significantly, newly formed habitats may temporarily contract due to water limitations. The actual accessibility of future suitable habitats requires integrated assessment incorporating the spatial distribution of soil texture. If the predicted areas are dominated by heavy clayey red soils, the actual establishment of *Plantago asiatica* may be severely constrained despite favorable climatic conditions.

### 3.4. Conservation and Cultivation Planning Recommendations

In situ conservation should focus on the current highly suitable habitats located in the central-northern transitional zone of Jiangxi (the border areas of Nanchang, Yichun, Xinyu, and Fuzhou). This region harbors the richest wild germplasm resources of *Plantago asiatica* in Jiangxi Province, but it also faces land development pressure from rapid urbanization and agricultural intensification. Urbanization constitutes a major threat to the highly suitable habitat patches in this region. These habitats, predominantly located in peri-urban areas, are highly prone to being occupied by construction land. Soil sealing would irreversibly destroy their loose soil structure with low clay content, making recovery challenging. Therefore, it is imperative to incorporate these areas into the urban ecological conservation redlines to safeguard the remaining suitable habitats. It is recommended that the current highly suitable habitat grids be designated as the core, with a 1–2 km outward expansion as a conservation buffer zone, incorporated into the ecological protection red line or urban green space system to prevent progressive encroachment by urban construction. This approach is consistent with Kokou et al. [47], who used MaxEnt in combination with Zonation to identify priority conservation areas.

This study found that the centroid consistently remained within the extremely small-scale area at the border of Xiajiang County (Mabu Town) and Xingan County (Maixie Town), and that highly suitable habitats did not exhibit significant expansion under any scenario. Therefore, it is recommended to establish a core cultivation base based on this stable core area. In addition, for regions exhibiting relative stability and moderate suitability, such as central-southern Jiangxi (Ji’an and Fuzhou), moderate expansion can be carried out in conjunction with introduction and domestication efforts. For specific site selection, it is recommended to prioritize contiguous highly suitable habitat grids under the SSP126 and SSP585 scenarios for small-scale introduction trials, following the optimized cultivation area assessment framework proposed by Nie et al. [48].

Areas projected to show contraction trends, such as southern Ji’an and southwestern Fuzhou, should establish long-term fixed monitoring plots to verify whether the predicted contraction indeed occurs. Specific cultivation sites should prioritize plots with low clay content and well-drained conditions. Issues related to clayey compaction or high acidity in Jiangxi red soils can be ameliorated through application of organic fertilizers and lime amendments.

### 3.5. Limitations and Future Perspectives

As a correlative niche-based prediction tool, the MaxEnt model is inherently unable to incorporate biotic interactions such as interspecific competition, pests and diseases, or anthropogenic harvesting processes; thus, the predictions reflect potential rather than actual distributions. As a synanthropic species, wild *Plantago asiatica* populations are subject to substantial harvesting pressure, a factor not represented in the model, potentially leading to overestimation of the actual accessibility of some highly suitable habitats. Although the 79 spatially rarefied occurrence records in this study fall within the acceptable range for MaxEnt modeling, the moderate sample size combined with the complex topography may lead to a conservative estimation of the species’ niche breadth. Therefore, further field surveys are required to validate and refine these predictions [49]. Additionally, the static soil assumption fails to capture the edaphic changes driven by ongoing urbanization and agricultural intensification. In future work, we plan to employ process-based soil evolution models to dynamically simulate the changes in soil texture and structure, while coupling dynamic models of dispersal limitations, thereby enhancing the model’s capacity to predict long-term habitat suitability. Subsequent studies need to integrate CMIP6 multi-model ensembles, refined environmental data, and field-based ecological information to reduce the uncertainty of extrapolation and thereby enhance the reliability of future predictions. The present study has answered the question of “where it is suitable to grow”; future work could measure the bioactive compound contents of *Plantago asiatica* from different suitable areas to construct quantitative quality–environment factor models, achieving a refined regionalization upgrade from “suitability” to “suitability + quality” [42,50]. Additionally, the quantitative relationships between the nonlinear characteristics of centroid migration and specific climatic factors warrant further in-depth analysis.

## 4. Materials and Methods

### 4.1. Study Area

**Figure 8:**
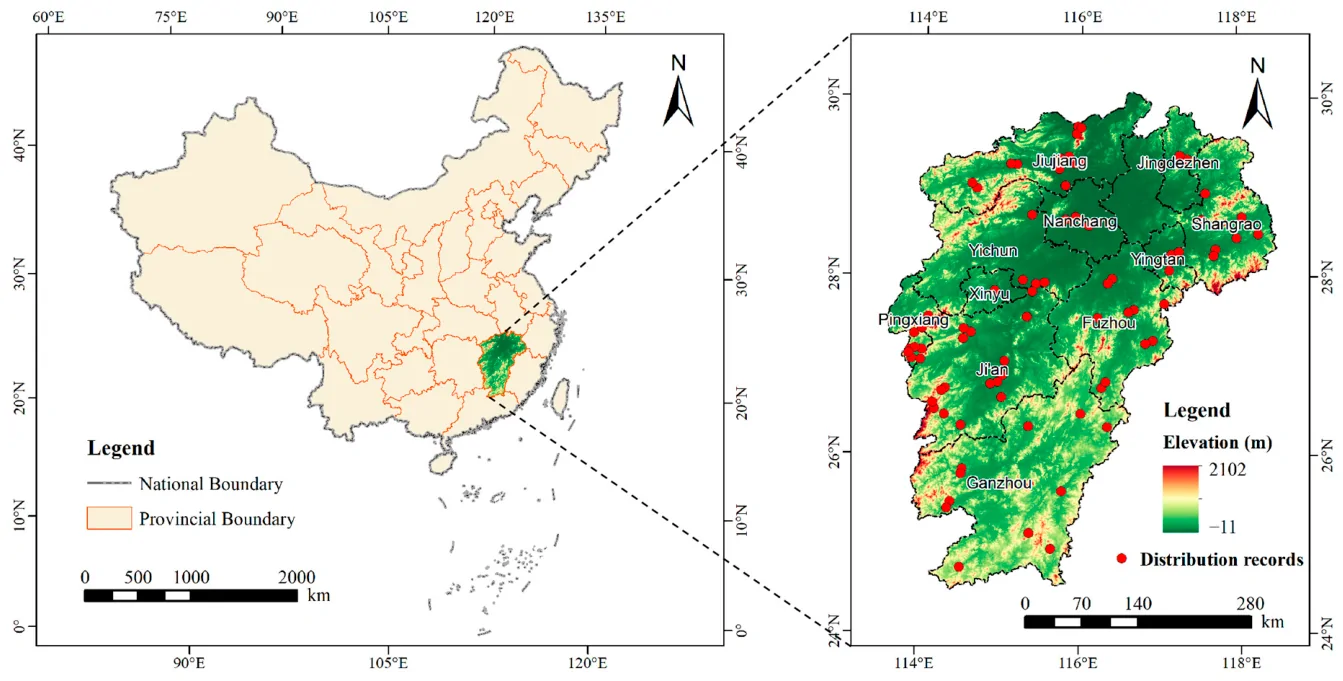
Map of the study area (elevation and distribution of *Plantago asiatica* in Jiangxi Province).

Jiangxi Province is located in southeastern China, on the southern bank of the middle and lower reaches of the Yangtze River, spanning 24°29′14″ N to 30°04′41″ N and 113°34′36″ E to 118°28′58″ E (Figure 8). The province has a subtropical humid monsoon climate, characterized by four distinct seasons, abundant sunshine, ample precipitation, and a long frost-free period. These mild and humid climatic conditions have given rise to diverse ecosystem types, providing a suitable habitat foundation for the growth of *Plantago asiatica*.

### 4.2. Data Source

#### 4.2.1. Occurrence Records of Plantago asiatica

Occurrence records of *Plantago asiatica* were obtained from the Global Biodiversity Information Facility (GBIF, https://www.gbif.org, accessed on 16 September 2025) and the Chinese Virtual Herbarium (CVH, http://www.cvh.ac.cn, accessed on 15 September 2025). Records with incomplete longitude/latitude information, duplicate entries, and those located in obviously unnatural habitats were first removed. To avoid model overfitting caused by spatially clustered occurrence points, a 1 km × 1 km grid was created in ArcGIS 10.8, and only one record was retained per grid cell, resulting in 79 validated occurrence points [51]. The longitude and latitude coordinates of the occurrence points were converted to decimal degrees and saved in .csv format as required by the MaxEnt software (version 3.4.4). Given the inconsistencies in spatial resolution and geographic coordinate systems among the environmental data, all raster data were first reprojected to the GCS_WGS_1984 geographic coordinate system using ArcGIS 10.8 software and subsequently converted to .asc format for MaxEnt modeling.

#### 4.2.2. Environmental Data

A total of 29 environmental variables from three categories—climate, topography, and soil—were selected for this study (Table 4). Climate data were sourced from the WorldClim database (http://www.worldclim.org, accessed on 15 September 2025), covering both current (1970–2000) and future (2021–2100) periods at a spatial resolution of 30 arc-seconds. Future climate data were derived from the BCC-CSM2-MR global climate model of the Coupled Model Intercomparison Project Phase 6 (CMIP6), under three shared socioeconomic pathway (SSP) scenarios—low (SSP126), medium (SSP370), and high (SSP585)—and were analyzed across four future time periods: 2021–2040, 2041–2060, 2061–2080, and 2081–2100. Topographic variables (elevation, slope, and aspect) were obtained from 30 m resolution Digital Elevation Model (DEM) data provided by the Geospatial Data Cloud. Soil data were sourced from the Harmonized World Soil Database version 2.0 (HWSD v2.0, https://gaez.fao.org/pages/hwsd, accessed on 15 September 2025), with an original resolution of 1 km, from which seven soil factors were selected.

### 4.3. Research Methods

#### 4.3.1. Pearson’s Correlation Analysis

To avoid model overfitting caused by multicollinearity among environmental variables, Pearson’s correlation analysis was performed on all environmental factors. Variable pairs with absolute correlation coefficients |r| ≥ 0.8 were identified as highly correlated pairs, and based on the contribution rates calculated from a preliminary MaxEnt model, the one with a higher contribution rate in each pair was retained [52,53].

#### 4.3.2. Maximum Entropy (MaxEnt) Model

The Maximum Entropy (MaxEnt, version 3.4.4) model is based on the principle of maximum entropy, which infers the potential distribution probability of a species within a study area using known occurrence points and environmental constraints. This model requires relatively small sample sizes and exhibits stable predictive performance, making it suitable for species distribution modeling at the regional scale of this study [54]. The complexity of the MaxEnt model is governed by the regularization multiplier (RM) and feature combinations (FC). The RM controls the smoothness of the model, while the FC includes linear (L), quadratic (Q), product (P), threshold (T), and hinge (H) features [55].

**Figure 9:**
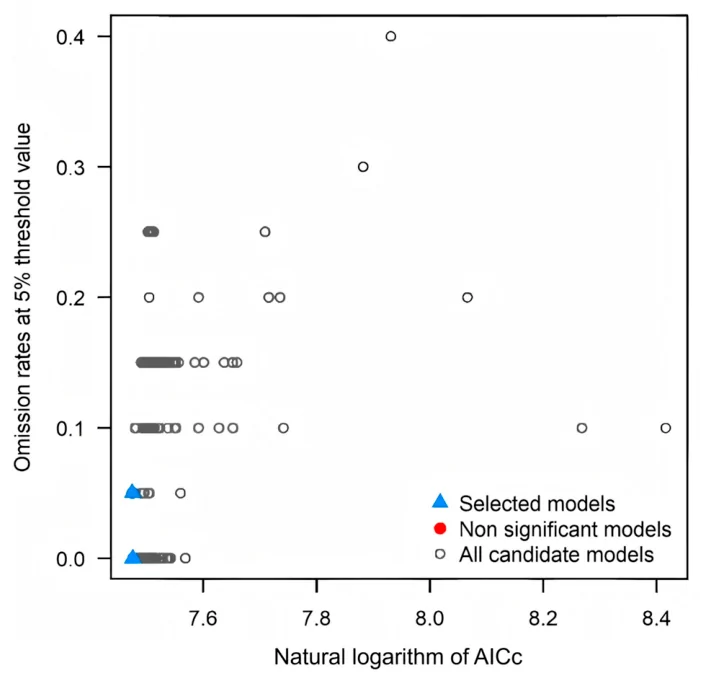
Results of optimal model selection based on ΔAICc.

The *kuenm* package in R was used to systematically optimize RM (ranging from 0.1 to 4.0 with a step of 0.1) and FC (31 combinations of L, Q, P, H, T, and their interactions). The optimal parameter combination was selected based on the minimum Akaike Information Criterion corrected for small sample sizes (ΔAICc = 0) and an omission rate < 5% [56,57]. The results identified FC = LQP and RM = 0.7 as the optimal combination (delta.AICc = 0), which achieved the best goodness-of-fit while reducing model complexity [58] (Figure 9).

The occurrence points (.csv) and environmental variables (.asc) were imported into MaxEnt 3.4.4. Ten-fold cross-validation was employed to train and test the model, and the output format was set to logistic, with all other parameters kept at their default values [59,60]. To reduce extrapolation risks, the multivariate environmental similarity surface (MESS) analysis built into MaxEnt was used to identify and exclude areas where environmental conditions exceeded the calibration range, thereby ensuring the reliability of the predictions.

#### 4.3.3. Centroid Function Method

The centroid function was used to describe the central position of the species distribution. The centroid coordinates (*x*, *y*) were calculated as the weighted average position of the species occurrence points [44,61]:

(1) $$x=\sum_{i=1}^{n}m_{i}x_{i}\;∕\;\sum_{i=1}^{n}m_{i}$$

(2) $$y=\sum_{i=1}^{n}m_{i}y_{i}\;∕\;\sum_{i=1}^{n}m_{i}$$

where *x* and *y* are the longitude and latitude of the centroid, *n* is the number of species occurrence points, *x_i_* and *y_i_* are the spatial coordinates of the *i*-th occurrence point, and *m_i_* is the weight assigned to the *i*-th occurrence point.

## 5. Conclusions

This study, employing a parameter-optimized MaxEnt model, identified the key environmental factors constraining the distribution of *Plantago asiatica*, predicted the evolutionary patterns of its suitable habitats under different climate scenarios, and proposed conservation and cultivation planning recommendations. The results indicated that the core constraining factor governing the regional distribution of *Plantago asiatica* is not climate, but soil clay content, whose influence exceeds the combined contribution of temperature, precipitation, and elevation factors. Currently, highly suitable habitats are highly concentrated in the central-northern transitional zone of Jiangxi, which is highly consistent with the actual layout of Jiangxi as a dao-di production area for *Plantago asiatica* in China. Under all three future SSP scenarios, the area of highly suitable habitats did not exhibit significant expansion, with its proportion consistently stable at 2.07–2.38% of the provincial area. The centroid consistently oscillated locally around the border zone of Mabu Town and Tonglin Township in Xiajiang County and Maixie Town in Xingan County. This prediction is based on the assumption that soil factors remain unchanged, reflecting the trend of climate-driven potential suitability. Based on these findings, it is recommended that the central-northern core region of Jiangxi be subject to strict development and construction controls and be incorporated into the ecological protection red line; that moderate introduction trials be conducted in the periphery of the core region and in relatively stable areas of central-southern Jiangxi; and that long-term monitoring plots be established in areas projected to show contraction trends, such as southern Ji’an and southwestern Fuzhou, to provide a basis for adaptive management of protected areas.

## Figures and Tables

**Table 1:** Contribution rates and permutation importance of the key environmental factors.

Environmental Factor	Contribution Rate (%)	Permutation Importance (%)	Environmental Factor	Contribution Rate (%)	Permutation Importance (%)
clay	56.7	26.3	bio8	3.0	0.8
bio3	12.4	14.9	bio14	2.8	18.3
alt	6.5	23.4	bio1	1.5	3.5
bio19	6.0	2.1	bio17	1.5	1.8
bio15	6.0	6.4	bio18	0.2	0.3
aspect	3.3	2.2			

**Table 2:** Area statistics of each suitability class for *Plantago asiatica* under different climate scenarios and periods.

Scenarios	Period	Highly Suitable		Moderately Suitable		Low Suitable		Unsuitable	
		Area/km^2^	Proportion	Area/km^2^	Proportion	Area/km^2^	Proportion	Area/km^2^	Proportion
Current		0.39 × 10^4^	1.40%	2.16 × 10^4^	14.77%	4.43 × 10^4^	34.51%	9.71 × 10^4^	49.32%
SSP126	2021–2040	0.40 × 10^4^	2.38%	2.21 × 10^4^	13.23%	4.30 × 10^4^	25.73%	9.79 × 10^4^	58.66%
	2041–2060	0.38 × 10^4^	2.26%	2.18 × 10^4^	13.04%	4.25 × 10^4^	25.45%	9.89 × 10^4^	59.25%
	2061–2080	0.38 × 10^4^	2.26%	2.15 × 10^4^	12.89%	4.33 × 10^4^	25.96%	9.83 × 10^4^	58.89%
	2081–2100	0.40 × 10^4^	2.38%	2.16 × 10^4^	12.92%	4.28 × 10^4^	25.61%	9.86 × 10^4^	59.09%
SSP370	2021–2040	0.38 × 10^4^	2.25%	2.09 × 10^4^	12.50%	4.19 × 10^4^	25.10%	10.04 × 10^4^	60.14%
	2041–2060	0.37 × 10^4^	2.23%	2.23 × 10^4^	13.36%	4.52 × 10^4^	27.10%	9.57 × 10^4^	57.31%
	2061–2080	0.36 × 10^4^	2.14%	2.22 × 10^4^	13.29%	4.28 × 10^4^	25.67%	9.83 × 10^4^	58.90%
	2081–2100	0.35 × 10^4^	2.10%	2.08 × 10^4^	12.49%	4.16 × 10^4^	24.94%	10.09 × 10^4^	60.47%
SSP585	2021–2040	0.39 × 10^4^	2.31%	2.07 × 10^4^	12.38%	4.12 × 10^4^	24.69%	10.12 × 10^4^	60.63%
	2041–2060	0.35 × 10^4^	2.07%	2.11 × 10^4^	12.63%	4.34 × 10^4^	25.97%	9.90 × 10^4^	59.33%
	2061–2080	0.37 × 10^4^	2.21%	2.10 × 10^4^	12.58%	4.21 × 10^4^	25.21%	10.01 × 10^4^	60.00%
	2081–2100	0.38 × 10^4^	2.28%	2.08 × 10^4^	12.46%	4.39 × 10^4^	26.29%	9.84 × 10^4^	58.97%

**Table 3:** Migration direction and distance of the distribution centroid of *Plantago asiatica* in Jiangxi Province across different periods.

Scenarios	Period	Longitude	Latitude	Distance Traveled (km)	Movement Direction
Current	Current	115.4411	27.5313	NA	NA
SSP126	2021–2040	115.4356	27.5760	4.98	North
	2041–2060	115.4722	27.5925	3.97	Northeast
	2061–2080	115.5093	27.6153	4.37	Northeast
	2081–2100	115.4082	27.5434	12.74	Southwest
SSP370	2021–2040	115.5153	27.6078	10.67	Northeast
	2041–2060	115.4346	27.5270	11.03	Southwest
	2061–2080	115.4914	27.5824	7.49	Northeast
	2081–2100	115.4830	27.5264	6.25	South
SSP585	2021–2040	115.4576	27.5311	1.63	East
	2041–2060	115.4861	27.4824	5.89	Southeast
	2061–2080	115.4892	27.5723	9.99	North
	2081–2100	115.4795	27.5628	1.40	Southwest

**Table 4:** Environmental variables used in this study.

Type	Code	Description	Unit	Data Source
Climate	bio1	Annual mean temperature	°C	WorldClim (http://www.Worldclim.org, accessed on 15 September 2025)
	bio2	Mean diurnal range	°C	
	bio3	Isothermality (bio2/bio7) × 100	—	
	bio4	Temperature seasonality (standard deviation × 100)	—	
	bio5	Max temperature of warmest month	°C	
	bio6	Min temperature of coldest month	°C	
	bio7	Temperature annual range (bio5-bio6)	°C	
	bio8	Mean temperature of wettest quarter	°C	
	bio9	Mean temperature of driest quarter	°C	
	bio10	Mean temperature of warmest quarter	°C	
	bio11	Mean temperature of coldest quarter	°C	
	bio12	Annual precipitation	mm	
	bio13	Precipitation of wettest month	mm	
	bio14	Precipitation of driest month	mm	
	bio15	Precipitation seasonality	—	
	bio16	Precipitation of wettest quarter	mm	
	bio17	Precipitation of driest quarter	mm	
	bio18	Precipitation of warmest quarter	mm	
	bio19	Precipitation of coldest quarter	mm	
Topographical	alt	Altitude	m	Geospatial Data Cloud (30 m DEM)
	aspect	Aspect	—	
	slope	Slope	°	
Soil	awc	Soil available water content	mm/m	HWSD v2.0 (https://gaez.fao.org/pages/hwsd, accessed on 15 September 2025)
	ph	pH	—	
	clay	Clay content	%wt.	
	sand	Sand content	%wt.	
	silt	Silt content	%wt.	
	org_carbon	Organic carbon content	%wt.	
	total_n	Total nitrogen content	g/kg	

## Data Availability

The original contributions presented in this study are included in the article. Further inquiries can be directed to the corresponding author.

## References

[1] Kirillov V., Massalova V., Pathak A., Ivashchenko A., Kabanova S., Rakhimzhanov A., Stikhareva T. (2025). Insights into Ex Situ Conservation and MaxEnt-Based Habitat Suitability Modeling of *Prunus ulmifolia* Franch. (Rosaceae Juss.): A Central Asian Relict Species. Trees For. People.

[2] Zhang T., Ma X., Li J., Li Y., Chen Q., Zhang C., Fu S. (2024). Climate Change Threatens the Distribution of *Liriodendron chinense*: Evidence from China. Clim. Serv..

[3] Luo T., Zheng B., Huang Y., Luo C., Zhang J., Liu Z., Wan W. (2025). Evaluation of Ecological Environment in Jiangxi Province Based on Improved Ecological Index. Remote Sens. Technol. Appl..

[4] Abrha H., Dodionom S., Ongoma V., Hagos H., Birhane E., Gebresamuel G., Manaye A. (2024). Response of Plant Species to Impact of Climate Change in Hugumbrda Grat-Kahsu Forest, Tigray, Ethiopia: Implications for Domestication and Climate Change Mitigation. Trees For. People.

[5] Wang L. (2024). A Review of Plantain Research. Spec. Econ. Anim. Plants.

[6] Chen B., Cao H., Lyu R., Wang L., Cheng X., Jin L. (2021). Study on Ecological Suitability Regionalization of Plantaginis Semen Based on MaxEnt and ArcGIS. Chin. J. Inf. Tradit. Chin. Med..

[7] Kong W., Li X., Zou H. (2019). Optimizing MaxEnt Model in the Prediction of Species Distribution. Chin. J. Appl. Ecol..

[8] Chen F., Li Y., Yang G. (2025). Analysis of Comprehensive Evaluation Index for Maize Cultivation Suitability in the Henan Southeast Based on GIS. Remote Sens. Technol. Appl..

[9] Zhang J., Wang S., Wang Z., Ma G. (2022). Fine Evaluation and Layout of Crop Suitability in Northern Shaanxi Based on GIS. Agric. Res. Arid. Areas.

[10] Xi W., Fu P. (2022). Land Suitability Evaluation of Citrus Cultivation Based on Fuzzy Mathematics. Acta Agric. Zhejiangensis.

[11] Weng Q., Chen W., Yuan Y., Shi H., Wang D., Li J., Ma Y., Yu Y. (2026). Suitability Evaluation of Honghuadajinyuan Tobacco in Dali Prefecture Based on Maximum Entropy Model. J. Southwest Univ. (Nat. Sci. Ed.).

[12] Xu Z., Peng H., Peng S. (2015). The Development and Evaluation of Species Distribution Models. Acta Ecol. Sin..

[13] Guisan A., Thuiller W. (2005). Predicting Species Distribution: Offering More than Simple Habitat Models. Ecol. Lett..

[14] Elith J., Graham C.H., Anderson R.P., Dudík M., Ferrier S., Guisan A., Hijmans R.J., Huettmann F., Leathwick J.R., Lehmann A. (2006). Novel Methods Improve Prediction of Species’ Distributions from Occurrence Data. Ecography.

[15] Ahmadi M., Hemami M.R., Kaboli M., Shabani F., Ashrafi S. (2023). MaxEnt Brings Comparable Results When the Input Data Are Being Completed; Model Parameterization of Four Species Distribution Models. Ecol. Evol..

[16] Tattoni C., Rizzolli F., Pedrini P. (2012). Can LiDAR Data Improve Bird Habitat Suitability Models?. Ecol. Model..

[17] Zhao J., Shao W., Li Y., Wang Y., Li D. (2024). Potential Impact of Climate Change on the Distribution of Capricornis milnewardusii, a Vulnerable Mammal in China. Ecol. Evol..

[18] Li Q., Qi Y., Wang Q., Wang D. (2022). Prediction of the Potential Distribution of Vaccinium uliginosum in China Based on the Maxent Niche Model. Horticulturae.

[19] Wang J., Ding H., Shao M., Yang F., Zeng J., Zeng F. (2023). Prediction of Potential Distribution Areas of Scaly-Sided Mergansers Based on MaxEnt Modeling in Jiangxi Province. Chin. J. Appl. Environ. Biol..

[20] Wu B., He G., Wang W. (2022). Prediction of Potentially Suitable Distribution Area of Yangtze Finless Porpoise in Jiangxi Province Based on MaxEnt Model. Chin. J. Wildl..

[21] Yang P., Zhao Z., Nie L., Xu W., Li Y., Wang L. (2025). Spatiotemporal Dynamics of Ecological Plantations of Camellia sinensis var. assamica: Synergistic Effects of Climate Change and Policy Constraints in Yunnan, China. Ind. Crops Prod..

[22] Juma I., Valencia J.B., Cortés A.J. (2026). Predicting Suitable Regions for Avocado (*Persea americana* Mill.) Tree Cultivation in Tanzania. Horticulturae.

[23] Zhao Y.H., Jiang L.M., Qiu L., Fu Q.L., Tan S.L. (2025). Climate-Driven “Nowhere to Go” for Alpine Plants: Impact of Climate Change on the Geographic Distribution of Dipsacoideae Species in China. Glob. Ecol. Conserv..

[24] Jia Y., Shu H., Yang Y., Li W., Lei G., Qi L. (2026). Northward Expansion and Southward Contraction of Potential Bamboo Forest Distribution in China under Future Climate Change Scenarios Based on the MaxEnt Model. Trees For. People.

[25] Dembélé J.B., Dimobe K., Konda B., Emmanuel Traoré I.C., Boussim I.J. (2025). Modelling the Current and Future Geographical Distribution of *Combretum glutinosum* Perr. ex DC. under Climate Change in Burkina Faso: Future Challenges and Conservation Opportunities. J. Nat. Conserv..

[26] Liao D., Zhou B., Xiao H., Zhang Y., Zhang S., Su Q., Yan X. (2025). MaxEnt Modeling of the Impacts of Human Activities and Climate Change on the Potential Distribution of *Plantago* in China. Biology.

[27] Ali M., Huang Z., Wani Z.A., Song J., Wu B., Hussain K., Rahim P.P.A., Wang J., Zhou F., Bussmann R.W. (2026). Conservation Deficits and Climate-Driven Habitat Shifts of Threatened Plants in South Asia. Geogr. Sustain..

[28] Cobos M.E., Peterson A.T., Barve N., Osorio-Olvera L. (2019). *Kuenm*: An R Package for Detailed Development of Ecological Niche Models Using Maxent. PeerJ.

[29] Wang E., Lu Z., Rohani E.R., Ou J., Tong X., Han R. (2024). Current and Future Distribution of *Forsythia suspensa* in China under Climate Change Adopting the MaxEnt Model. Front. Plant Sci..

[30] Zeng J., Li C., Liu J., Li Y., Hu Z., He M., Zhang H., Yan H. (2021). Ecological Assessment of Current and Future *Pogostemon cablin* Benth. Potential Planting Regions in China Based on MaxEnt and ArcGIS Models. J. Appl. Res. Med. Aromat. Plants.

[31] Gebrewahid Y., Abrehe S., Meresa E., Eyasu G., Abay K., Gebreab G., Kidanemariam K., Adissu G., Abreha G., Darcha G. (2020). Current and Future Predicting Potential Areas of *Oxytenanthera abyssinica* (A. Richard) Using MaxEnt Model under Climate Change in Northern Ethiopia. Ecol. Process..

[32] Zhou W., Li B., Xu H., Liang Z., Lu X., Yang L., Wang W. (2023). Potential Distribution of Two Economic Laver Species—*Neoporphyra haitanensis* and *Neopyropia yezoensis*—Under Climate Change Based on MaxEnt Prediction and Phylogeographic Profiling. Ecol. Indic..

[33] Wang X., Li Z., Zhang L., Wang Y., Liu Y., Ma Y. (2024). The Optimized Maxent Model Reveals the Pattern of Distribution and Changes in the Suitable Cultivation Areas for *Reaumuria songarica* Being Driven by Climate Change. Ecol. Evol..

[34] Elith J., Phillips S.J., Hastie T., Dudík M., Chee Y.E., Yates C.J. (2011). A Statistical Explanation of MaxEnt for Ecologists. Divers. Distrib..

[35] Phillips S.J., Anderson R.P., Dudík M., Schapire R.E., Blair M.E. (2017). Opening the Black Box: An Open-source Release of Maxent. Ecography.

[36] Guo Y., Zhang S., Ren L., Tian X., Tang S., Xian Y., Wu X., Zhang Z. (2024). Prediction of Chinese Suitable Habitats of *Panax notoginseng* under Climate Change Based on MaxEnt and Chemometric Methods. Sci. Rep..

[37] Guo Y., Zhang S., Tang S., Pan J., Ren L., Tian X., Sun Z., Zhang Z. (2023). Analysis of the Prediction of the Suitable Distribution of *Polygonatum kingianum* under Different Climatic Conditions Based on the MaxEnt Model. Front. Earth Sci..

[38] Bai J., Wang H., Hu Y. (2024). Prediction of Potential Suitable Distribution of *Liriodendron chinense* (Hemsl.) Sarg. in China Based on Future Climate Change Using the Optimized MaxEnt Model. Forests.

[39] Liang J., Tang G., Qin X. (2024). Delineating the Area for Sustainable Cultivation of *Morinda officinalis* Based on the MaxEnt Model. Agronomy.

[40] Liu H., Gong H., Qi X., Li Y., Lin Z. (2018). Relative Importance of Environmental Variables for the Distribution of the Invasive Marsh Species Spartina alterniflora across Different Spatial Scales. Mar. Freshw. Res..

[41] Wang Z., Liu H., Chen P., Li J., Wang B., Zi S., Duan Q. (2026). Effects of Pavement Excavation on Root Characteristics of Four Typical Plant Species in Subalpine Meadow. Bull. Soil Water Conserv..

[42] Wu D., Qin Q., Xu C., Chen M., Liu M., Wu Y., Zhang S. (2024). Analysis of Suitable Areas and Quality Regionalization of *Ligusticum sinense* cv. Chaxiong in Jiangxi Province. J. Chin. Med. Mater..

[43] Ren W., Peng J., Shrestha N., Bian Z., Yang Y., Liu J., Liu X., Huang P., Wu J. (2025). Potential Distribution and Future Shifts of Invasive Alien Plants in China under Climate Change. Glob. Ecol. Conserv..

[44] Tian Y., Song J., Cheng B., Wei R., Zeng Y., Zhang J., Zhang J., Wang Z. (2025). Modeling the Impact of Climate Change on the Distribution of *Populus adenopoda* in China Using the MaxEnt Model. Forests.

[45] Gómez-Ruiz E.P., Lacher T.E. (2019). Climate Change, Range Shifts, and the Disruption of a Pollinator-Plant Complex. Sci. Rep..

[46] Cai D., Wang S., Li W., Wang K., Zhu G., Zhang Z., Qu S., Liu Y. (2026). Projecting the Potential Shift of *Larix principis-rupprechtii* in Response to Future Climate Change: A Regional Analysis of the Haihe Basin in Northern China. Forests.

[47] Kokou B.K., Ulemu M., Atakpama W., Biaou S., Teteli S.C., Sambieni K.R., Mnthambala F., Mavuto T., Munyenyembe P., Noulèkoun F. (2025). Climate Change Reduces and Shifts Suitable Habitats of *Uapaca kirkiana Müll. Arg.* to Higher Altitudes in Malawi. Trees For. People.

[48] Nie Y., Yu G., Hu H. (2025). Optimal Planting Areas of Sea Buckthorn (*Hippophae rhamnoides*) Under the Influences of Climate Change and Pests Using the MaxEnt Model. Agronomy.

[49] Xu W.B., Svenning J.C., Chen G.K., Zhang M.G., Huang J.H., Chen B., Ordonez A., Ma K.P. (2019). Human activities have opposing effects on distributions of narrow-ranged and widespread plant species in China. Proc. Natl. Acad. Sci. USA.

[50] Yin X., Qiao Y., Liu Y., Song X., Han S., Wang J. (2026). Study on the Suitability Regionalization and Environmental Factors of *Centipeda minima* Based on MaxEnt. J. Chin. Med. Mater..

[51] El Haddouti I., Karmoudi Y.E., Khabbach A., Libiad M. (2026). Predicting the Distribution of *Taxus baccata* L. in Morocco Under Climate Change Using MaxEnt: Implications for Conservation and Sustainable Management. Sustainability.

[52] Bakar A.H.A., Hassan M.N.M., Zakaria A., Ashraf A.H.A. (2019). Pearson’s Correlation Coefficient Analysis of Non-Invasive Jaundice Detection Based on Colour Card Technique. J. Phys. Conf. Ser..

[53] Pan S., Liu Z., Han Y., Zhang D., Zhao X., Li J., Wang K. (2024). Using the Pearson’s Correlation Coefficient as the Sole Metric to Measure the Accuracy of Quantitative Trait Prediction: Is It Sufficient?. Front. Plant Sci..

[54] Huang T., Yang T., Wang K., Huang W. (2024). Assessing the Current and Future Potential Distribution of *Solanum rostratum* Dunal in China Using Multisource Remote Sensing Data and Principal Component Analysis. Remote Sens..

[55] Xu Z., Huang T., Dai L., Huang F., Gao H. (2026). Potential Occurrence Area Prediction of Pine Wilt Disease in Xinjiang (Northwestern China) by Maximum Entropy Model. Forests.

[56] Wang H., Liu Q., Shen J., Ding J., Zeng Y., Zhou Z., Yan X., Zhang J., Ma X., Yu Q. (2025). Modeling the Future Distribution of Trifolium repens L. in China: A MaxEnt Approach Under Climate Change Scenarios. Biology.

[57] Liu Q., Charleston M.A., Richards S.A., Holland B.R. (2023). Performance of Akaike Information Criterion and Bayesian Information Criterion in Selecting Partition Models and Mixture Models. Syst. Biol..

[58] Bao R., Li X.L., Zheng J.H. (2022). Feature Tuning Improves MAXENT Predictions of the Potential Distribution of *Pedicularis longiflora* Rudolph and Its Variant. PeerJ.

[59] Tong L., Wu Z., Huang W., Hu M., Liu S., Han Y., Zhang G., Ye Y. (2026). Modeling the Potential Distribution and Spatial Dynamics of *Chenopodium hybridum* in China Under Climate Change and Human Disturbance. Diversity.

[60] Kass J.M., Muscarella R., Galante P.J., Bohl C.L., Pinilla-Buitrago G.E., Boria R.A., Soley-Guardia M., Anderson R.P. (2021). ENMeval 2.0: Redesigned for Customizable and Reproducible Modeling of Species’ Niches and Distributions. Methods Ecol. Evol..

[61] Jiang H., Chen H., Wang H., Huang B., Chen T. (2025). Establishment of Integrated Quality Regions for the Rare Medicine Food Homology Plant *Cyclocarya paliurus* (Batal.) Iljinsk in China. Biology.

